# A randomized controlled trial to evaluate a behavioral economic strategy for improving mobility in veterans with chronic pain

**DOI:** 10.1371/journal.pone.0257320

**Published:** 2021-10-11

**Authors:** Peggy Compton, Krisda H. Chaiyachati, Tanisha Dicks, Elina Medvedeva, Manik Chhabra

**Affiliations:** 1 School of Nursing, University of Pennsylvania, Philadelphia, Pennsylvania, United States of America; 2 The Leonard Davis Institute of Health Economics at the University of Pennsylvania, Philadelphia, Pennsylvania, United States of America; 3 Department of Medicine, The Perelman School of Medicine at the University of Pennsylvania, Philadelphia, Pennsylvania, United States of America; 4 Department of Veterans Affairs Center for Health Equity Research and Promotion (CHERP) Philadelphia, Pennsylvania, United States of America; Prince Sattam Bin Abdulaziz University, College of Applied Medical Sciences, SAUDI ARABIA

## Abstract

Rates of chronic pain and daily opioid use are higher among veterans relative to civilian populations. Increasing physical activity can reduce pain severity and decrease opioid use among patients with chronic pain. Behavioral economic strategies can improve physical activity levels but have been undertested in veterans with chronic pain. The objective of this study was to evaluate if a financial incentive combined with a loss aversion component—a “regret lottery” in which veterans could win money if they met a set goal or told how much they could have won had they met their goal—would increase physical activity levels among veterans with chronic pain. A 12-week single-blinded randomized controlled trial (ClinicalTrials.gov: NCT04013529) was designed. Veterans with chronic pain (N = 40) receiving care at a specialty pain clinic were eligible for participation, and were randomly assigned (1:1) to either (a) activity trackers and daily text message reminders to increase physical activity (“control arm”), or (b) the same plus a weekly regret lottery (“intervention arm”). For those in the intervention arm, participants who met their activity goal, had a chance to win a small ($30) or large ($100) gift card incentive; those who did not meet their goals were informed of what they would have won had they met their goal. The primary outcome, physical activity, was measured using self-reported physical activity and step counts using activity trackers. Secondary outcomes included changes in physical function, chronic pain severity, depression and opioid use. The sample was primarily white, male and disabled, with an average age of 57 years. No between-arm differences were noted for physical activity, physical function, chronic pain severity, depression or opioid use. Regret lottery-based approaches may be ineffective at increasing physical activity levels in veterans with chronic pain.

Trial Registry: NCT04013529.

## Introduction

Chronic pain, defined as pain that persists or recurs for more than 3 months [1], is a highly prevalent and costly condition in the United States. An estimated 88.5 million adults suffer from chronic pain [2], resulting in $500–635 billion lost due to less productivity and $261–300 billion in additional health care expenditures [3]. Chronic pain is particularly common within the veteran population [4, 5], for whom, the prevalence of low back pain is increasing more rapidly than depression or diabetes [6], and the prevalence of musculoskeletal pain increases in the years following deployment [7]. Rates of regular opioid analgesic use among veterans are also high [8], putting them at increased risk for overdose, abuse and diversion [9–11]. Opioids are less effective in treating chronic pain compared with non-opioid approaches [12, 13], and current Centers for Disease Control and Veterans Affairs (VA)/Department of Defense (DOD) guidelines recommend non-opioid approaches for treating patients with chronic pain, and tapering patients on opioids to lower, safer doses or to none at all [14–16].

Increasing physical activity has been shown to improve pain and decrease medication use among patients with chronic pain [17–21]. A recent meta-analysis of Cochrane Reviews found exercise and physical activity improved pain severity, physical function, and quality of life for patients with chronic pain [22]. However, increasing physical activity in veterans can be challenging due to high rates of impeding medical and psychiatric comorbidities (e.g., heart disease, obesity, depression) [23–27].

Behavioral economic strategies that combine lottery incentives with a loss aversion component have been effective in promoting physical activity in non-veteran populations who do not have chronic pain [28–31], leveraging behavioral economic principles which posit that individuals are motivated by immediate gratification and will avoid feelings of regret. Lottery incentives combined with loss aversion strategies are more effective than either alone for promoting physical activity [32]. One such effective strategy is a regret lottery where participants can win money if they meet a predefined goal or are told how much they could have won, had they met their goal [33]. A regret lottery has been undertested among veterans with chronic pain and, if successful in motivating physical activity, may have significant impacts on subsequent pain-related outcomes.

The primary objective of this study was to determine if a weekly regret lottery could improve physical activity among veterans with chronic pain. The study’s secondary objectives were to explore if the lottery intervention would change physical function, chronic pain severity, depression, and opioid use.

## Methods

### Design

A single-blind randomized controlled trial was designed to evaluate the efficacy of a regret lottery to increase physical activity and pain-related outcomes in a population of veterans with chronic pain; although patients were not aware of their group assignment, the data collectors were. Data were collected between July 23, 2019 and January 24^th^, 2020. The Corporal Michael J. Crescenz VA Medical Center (CMC VAMC) in Philadelphia, Pennsylvania Human Studies Research & Development Committee approved the study (ClinicalTrials.gov: NCT04013529). The authors confirm that any ongoing and related trials for this intervention will be registered.

### Sample and setting

The sample was comprised of 40 veterans with chronic non-malignant pain receiving care from a pain-focused primary aligned care team (“Pain PACT”) at the CMC VAMC; being a pilot study, sample size was not calculated for effect, but to determine effect. Utilizing the approach outlined by Bell and colleagues (2018), a sample size of 20 per arm was deemed adequate to detect a small target effect size (0.1 < d < 0.3) in 80% powered main trial, or a medium effect size (0.3 < d < 0.7) in 90% powered main trial [34]. The Pain PACT provides comprehensive, evidence-based care for veterans with chronic pain not due to malignancy using a multidisciplinary team consisting of primary care providers, physical therapists, behavioral psychologists, social workers, pharmacists, and nurses. Patients are referred into the team from other primary care or subspecialty providers and are invited to enroll in the Pain PACT if they meet one or both of the following criteria: being on opioid therapy or having been diagnosed with a substance use disorder that is either active or in remission. Patients referred to the team may have either chronic primary pain, or chronic secondary pain that is not cancer related. To be eligible for study participation, patients had to have been a patient in Pain PACT within the past year and possess either an iPhone or Android cell phone. The original eligibility for the study was for patients only on opioid doses over 100 morphine milliequivalents daily; this was expanded to all patients in the Pain PACT regardless of current pain regimen as the PACT team did not have enough patients who met that dose criterion. Patients were excluded if they had visual impairments preventing the use of text messaging or the study’s activity tracker, or a physical disability precluding improvements in walking (e.g., wheelchair bound). Participants in both arms received $75 for complete study participation.

### Measures

#### Primary outcome

The primary study outcome was physical activity. Physical activity was assessed weekly using two measures: (1) the number of steps recorded using a Fitbit activity tracker, and (2) the Stanford 7-Day Physical Activity Recall Questionnaire (PAR) [35], a reliable and valid measure [36, 37] of time spent engaged in stretching/strengthening activities and aerobic activities. There was no specific physical activity program prescribed, rather the incentives were aimed at simply increasing physical activity in general. Patients could individualize the type and frequency of exercise.

#### Secondary outcomes

Secondary measures were pain-related physical function, chronic pain severity, symptoms of depression, and opioid analgesic use; these data were collected at baseline and at the completion of the 12-week study. Pain-related physical function was measured by the Pain Outcomes Questionnaire [38] which assessed the degree to which patients had chronic pain that (1) interfered with walking and completing activities of daily living, (2) affected feelings of vitality, (3) contributed to a negative affect, and (4) elicited fears of injury or re-injury; all were measured on a scale of 0 (not at all) to 10 (all the time). Chronic pain severity was also captured on the same tool, where participants were asked to rate the severity of their chronic pain on a scale of 0 (no pain at all) to 10 (worst possible pain).

Symptoms of depression were measured by the Patient Health Questionnaire-9 (PHQ-9), which queries patients on nine symptoms of depression over the past two weeks and rates each symptom question on a scale of 0 (not at all) to 4 (nearly every day), thus total score ranges from 0 to 36 [39]. Among the subset of patients prescribed an opioid-containing analgesic, baseline and the end of study opioid prescriptions were extracted from the electronic health record (formulation, dose, and quantity) for the previous 24-hours. Table 1 lists all study measures with frequency and mode of administration. In addition, participants’ demographics (race, gender, age, education level, marital status, living situation, mode of transportation, employment status and income) were collected at baseline (see S1 Table).

**Table 1:** Study measures.

Measure	Frequency	Range	Administration
Steps (Fitbit)	Weekly		Fitbit synced daily; weekly total entered analysis
Physical Activity Recall Questionnaire (PAR):	Weekly		Delivered via text message
Stretching/strengthening		0–180	
Aerobic		0–180	
Pain Severity (Pain Outcomes Questionnaire)	Baseline, 12 weeks	0–10	Delivered via text message
Pain Interference (Pain Outcomes Questionnaire):	Baseline, 12 weeks		Delivered via text message
Interference with walking		0–10	
Interference with ADLs		0–10	
Feelings of vitality		0–10	
Negative affect		0–10	
Fears of injury or re-injury		0–10	
Depression Symptoms (PHQ-9)	Baseline, 12 weeks	0–36	Delivered via text message
Daily Opioid Use	Baseline, 12 weeks		Extracted from electronic health record

ADLs–activities of daily living, PAR–Physical Activity Recall, PHQ-9–Patient Health Questionnaire-9; for statistical analyses, chi-square and Fisher’s exact tests were used for categorical variables, and Wilcoxon and t-tests were used for continuous variables.

### Procedures

Patients were recruited for participation by the Pain PACT staff and posted flyers. One co-author (MC) is the medical director of the clinic but was blinded to the randomization. Following provision of IRB-approved informed consent, participants were randomized 1:1 to (a) activity trackers and daily text message reminders to increase physical activity (“control arm”), or (b) the same plus a weekly regret lottery (“intervention arm”). Both arms were encouraged to meet a weekly activity goal of either (a) a 5% increase in steps over the previous week, or (b) achieving and maintaining the number of steps at 150% over baseline; no specific exercise program was prescribed. Mirroring prior studies [28, 40, 41], the regret lottery provided participants in the intervention arm who met either activity goal the weekly chance of winning a small ($30; 18% chance of winning) or large ($100; 1% chance of winning) incentive. Participants in the intervention arm who did not meet their goal were informed what they would have won had they met their goal. Both arms also received a daily text message reminding the subject to engage in physical activity and to achieve their weekly goals (see Table 2).

**Table 2:** Text messages.

Type	Message	Frequency
Welcome	Welcome to the study!	Once
	We will be contacting you at least daily with text reminders and messages to collect data about your physical activity and medication use.	
	To upload your Fitbit data, open the app every day to sync your device. This will send us your data! Remember to charge your Fitbit every two days.	
	Your average steps for the last 2 weeks was {{@Baseline}}, your target steps for the next week is {{@5% of baseline}}.	
Daily Reminder	Good morning! We hope you will reach your physical activity goals today!	Daily
Daily Reminder	Good evening! We hope you had a good day and were able to meet your activity goals today. Click Here SURVEY_LINK to enter your pain data.	Daily
Survey Reminder	Click Here SURVEY_LINK to enter your exercise data.	Weekly
Survey Reminder	Click here SURVEY_LINK to upload your health data! (Depression)	Monthly
Fitbit Upload	Fitbit upload: Remember to charge your Fitbit AND sync your Fitbit daily by opening the app on your phone! This is important because syncing your device sends us your data!	Daily
	*If you have met your activity goals*, *you will be entered in the lottery*, *and we will let you know if you have won weekly*!	
Step Goal Messages	On average this week, you walked {{@Weekly Average}} steps/day, which is good, but did not meet the goal of increasing your number of steps by 5% this week. Next week, try and meet your goal of {{@Target value}} steps.	Weekly
Step Goal Messages	Congratulations! This week you walked {{@Weekly Average}} steps/day–which met your goal of a 5% increase in steps this week.	Weekly
	*As a result*, *you will be entered into the lottery*! *Next week*, *try and meet your new goal of {{@5% above weekly average}} steps for another chance to win $30 or $100*!*”*	
Step Goal Messages	Congratulations! This week you walked {{@Weekly Average}} steps/day–which met your goal in steps this week.	Weekly
	*As a result*, *you will be entered into the lottery*! *Next week*, *try and walk {{@150% of Baseline}} steps again for another chance to win $30 or $100*!*”*	
Step Goal Messages	On average this week, you walked {{@Weekly Average}} steps/day, which is good, but did not meet the goal of increasing your number of steps by 5% this week.	Weekly
	*Thus*, *you were not entered in the lottery*. *If you were in the lottery you might have won $30 or $100*. *Next week*, *try and meet your goal of {{@Target value}} steps for another chance to win $30 or $100*!	
Lottery Messages	*LOTTERY RESULT*: *Congratulations*! *You won AMOUNT in the lottery*. *Your number was YOUR_NUMBER and the winning number was WINNING_NUMBER*.	Weekly
Lottery Messages	*LOTTERY RESULT*: *Unfortunately*, *you did not win the lottery this time*. *Your number was YOUR_NUMBER and the winning number was WINNING_NUMBER*.	Weekly
Lottery Messages	*LOTTERY RESULT*: *Had you met your goals*, *you would have won AMOUNT in the lottery*. *Your number was YOUR_NUMBER and the winning number was WINNING_NUMBER*.	Weekly
Lottery Messages	*LOTTERY RESULT*: *You would not have won the lottery this time had you been eligible*. *Your number was YOUR_NUMBER and the winning number was WINNING_NUMBER*.	Weekly

Note: Italicized content was sent only to those randomized to the regret lottery arm.

All participants were provided an activity tracker and verbal instructions on how to use the device and synchronize data uploads by a study team member (TD) who also provided device support throughout the study. To determine participants’ baseline physical activity level, tracker step data were collected for two-weeks prior to the patient receiving text messages specific to the assigned study arm. To encourage data collection in both arms, a text message was sent at the end of each day reminding subjects to sync their devices and directing them to complete the daily or weekly survey components. Online enrollment, randomization, bi-directional text messaging, the regret lottery, and integration of activity tracker data were all administered via the Way to Health platform, a research and clinical protocol management system housed at the University of Pennsylvania Center for Health Incentives and Behavioral Economics and Penn Medicine’s Center for Health Care Innovation (www.waytohealth.org) [42].

### Data analysis

We summarized the subject demographics and the study outcomes overall and by intervention arms at baseline and 12 weeks. The following study outcomes were included: activity tracker steps, activity tracker distance, weekly physical activity scores, pain severity, function, and opioid use. Categorical variables were summarized by N, %, and continuous variables were summarized by Mean (SD). With respect to opioid use, many of the subjects were taking buprenorphine formulated with daily naloxone for which morphine milligram equivalent (MME) conversion formulas are not available [15], therefore the daily buprenorphine dose was calculated and analyzed separately from other opioids. Demographics and baseline data were inspected to assess randomization. For each primary and secondary outcome, we tested the differences in baseline scores using chi-square and Fisher’s exact tests for categorical variables and Wilcoxon and t-tests for continuous variables.

We calculated the proportion of subjects achieving step goals for each week and summarized the overall numbers by Mean (SD) for each arm. We also conducted the Cochran-Armitage trend test to assess the change in proportions over time. Absolute change and percent change in scores between baseline and the follow up score at 12 weeks were calculated for all primary and secondary measures. Comparisons between the intervention and control arms were based on analysis of covariance general linear models. For each primary and secondary outcome, the baseline measure was used as a covariate, and the follow up score at 12 weeks was used as the dependent variable. All analyses were completed with SAS version 9.4 software.

## Results

The results outlined below address the primary objective of this study which was to determine if a weekly regret lottery could improve physical activity among veterans with chronic primary pain. In addition, the results describe how the lottery intervention changed physical function, chronic pain severity, depression and opioid use, thereby addressing the secondary objectives of the study.

**Fig 1:**
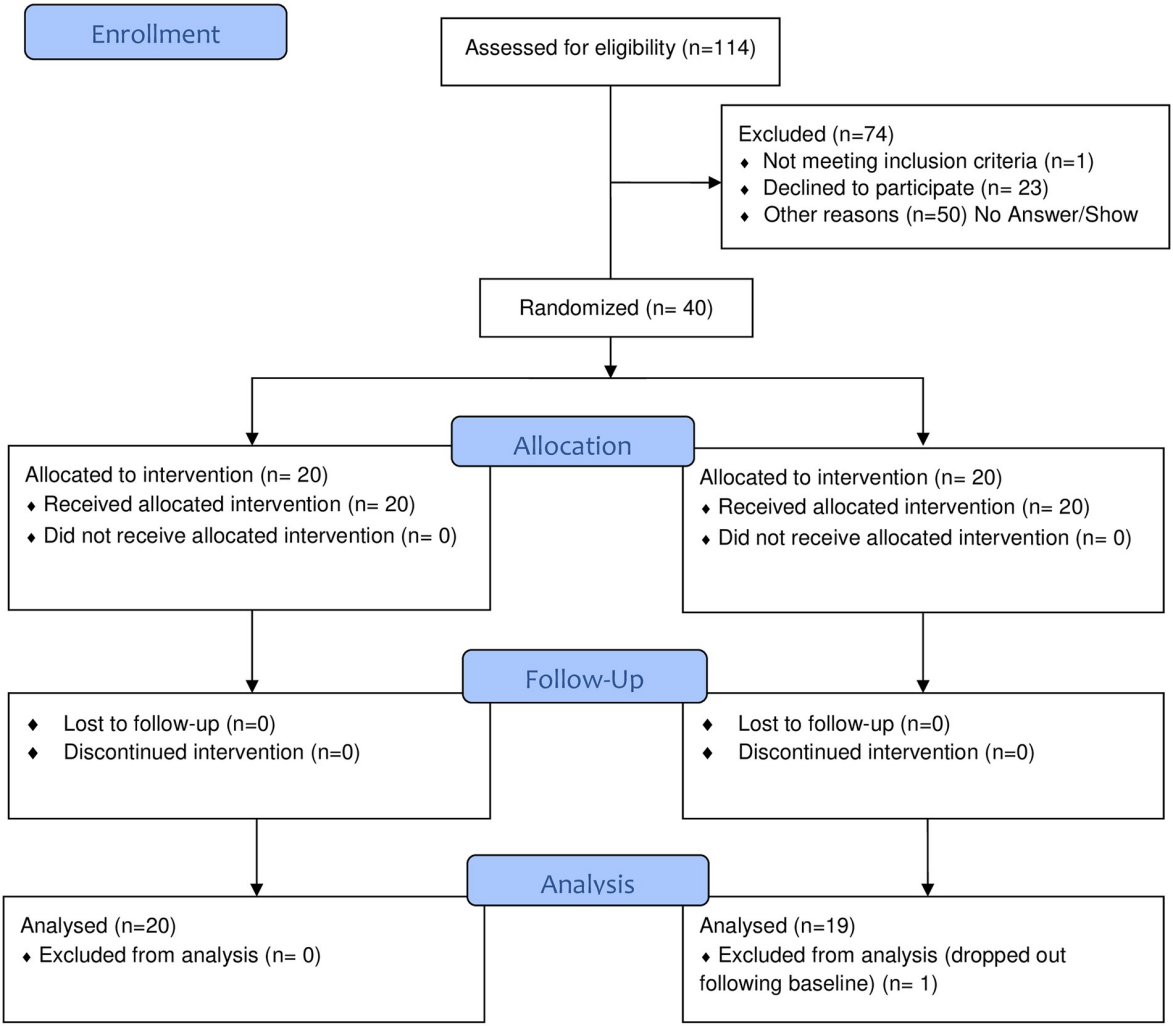
CONSORT 2010 flow diagram.

One-hundred and fourteen patients were approached for participation; the majority either did not respond to the invitation or declined to participate (see Fig 1). The 40 veterans who enrolled in the study were primarily male, middle-aged, White, not married, and had completed high school or obtained their GED (Table 3). Half were disabled and unable to work. Study participants primarily had back pain (34 of 40), with a history of degenerative disk disease, spinal stenosis, and/or radiculopathy. Three primarily had an inflammatory arthritis (rheumatoid, lupus, or psoriatic arthritis); two had a diagnosis of fibromyalgia; and, one with shoulder osteoarthritis. All patients also had other co-occurring chronic disease conditions, including at least one diagnosed mood disorder or post-traumatic stress disorder (37 of 40), and either a history of, or active, substance use disorder (32 of 40).

**Table 3:** Sample characteristics.

	**Total**		**Arm**				
			**Control**		**Intervention**		
	**N**	**Percent**	**N**	**Percent**	**N**	**Percent**	**P-values**
**Total**	40	100.0	20	100.0	20	100.0	
**Race**	15	37.5	8	40.0	7	35.0	0.80
**Black**							
**Other**	3	7.5	2	10.0	1	5.0	
**White**	22	55.0	10	50.0	12	60.0	
**Gender**	3	7.5	1	5.0	2	10.0	1.00
**Female**							
**Education**	16	40.0	9	45.0	7	35.0	0.22
**Above high school**							
**High school**	13	32.5	4	20.0	9	45.0	
**Less than high school**	3	7.5	1	5.0	2	10.0	
**Other**	8	20.0	6	30.0	2	10.0	
**Marriage status**	17	42.5	5	25.0	12	60.0	0.09
**Divorced or separated**							
**Married**	18	45.0	11	55.0	7	35.0	
**Never married**	5	12.5	4	20.0	1	5.0	
**Living situation**	14	35.0	5	25.0	9	45.0	0.19
**Live alone**							
**Live with others**	26	65.0	15	75.0	11	55.0	
**Transit situation**	36	90.0	19	95.0	17	85.0	0.23
**Driving**							
**Public transportation**	3	7.5	.	.	3	15.0	
**Shared van**	1	2.5	1	5.0	.	.	
**Hispanic**							1.00
**No**	35	87.5	17	85.0	18	90.0	
**Yes**	5	12.5	3	15.0	2	10.0	
**Employment**	20	50.0	11	55.0	9	45.0	0.27
**Disabled, not able to work**							
**In school**	1	2.5	1	5.0	.	.	
**Other**	1	2.5	.	.	1	5.0	
**Retired**	9	22.5	2	10.0	7	35.0	
**Unemployed**	3	7.5	2	10.0	1	5.0	
**Working**	6	15.0	4	20.0	2	10.0	
**Income**	4	10.0	2	10.0	2	10.0	0.27
**Above 30K**							
**Below 30K**	4	10.0	2	10.0	2	10.0	
**Do not know**	2	5.0	.	.	2	10.0	
**Refuse to disclose**	30	75.0	16	80.0	14	70.0	
**Travel time**	12	30.0	5	25.0	7	35.0	0.65
**15–30 minutes**							
**31–60 minutes (1 hour)**	16	40.0	9	45.0	7	35.0	
**61–90 minutes**	10	25.0	4	20.0	6	30.0	
**91–120 minutes (2 hours)**	1	2.5	1	5.0	.	.	
**Less than 15 minutes**	1	2.5	1	5.0	.	.	
**All care at VA**	3	7.5	1	5.0	2	10.0	1.00
**No**							
**Yes**	37	92.5	19	95.0	18	90.0	
**Doctors outside VA**	37	92.5	19	95.0	18	90.0	1.00
**No**							
**Yes**	3	7.5	1	5.0	2	10.0	
		**Mean (SD)**		**Mean (SD)**		**Mean (SD)**	
**Age**	40	57.4 (12.3)	20	55.2 (11.0)	20	59.6 (13.5)	0.28
**Number of people living with**	40	1.8 (1.9)	20	2.1 (1.9)	20	1.6 (1.9)	0.35
**Highest school grade completed**	40	13.1 (1.7)	20	13.5 (1.9)	20	12.8 (1.4)	0.16

Ninety-five percent (38 of 40) of participants completed the 12-week study period. During the intervention period, missing step data represented 4.6% (11 of 240 participant-weeks) of observations in the control arm and 5.0% (12 of 240 participant-weeks) of observations in the intervention arm. The mean (SD) unadjusted proportions of participant-weeks that step goals were achieved during the study period were 0.31 (0.09) in the control arm and 0.29 (0.14) in the intervention arm. The proportion of participants achieving step goals did not change throughout the study period in the control arm but decreased in the intervention arm (Cochran-Armitage trend test one-sided p-value = 0.03).

Of the 20 participants randomized to the intervention arm, 14 (70%) met their activity goals at least once during the 12-week trial, and thus were eligible for a lottery incentive. Fourteen participants (70%) received payment at least once during the trial, 13 (65%) of whom won $30 at least once (range 1–4 times). Three participants (15%) won $100 at least once (range 1–2 times); two (10%) of whom won both $30 and $100 during the trial. Notably, four of the lottery winners (20%) never claimed their winnings. Of the 10 participants (50%) who claimed their winnings, they earned between $30 and $300. Sixteen of the participants (80%) in the intervention arm received a regret message detailing what they would have won had they achieved their goal. Fifteen participants (75%) received a $30 regret message at least once during the trial (range 1–6 times). Three participants (15%) received a $100 regret message at least once (range 1–2 times), two (10%) of whom received at least one $30 and $100 regret message during the trial. No adverse events were experienced over the course of the study.

### Primary outcome—Physical function

The mean (SD) baseline daily step count was 5907 (3961.0) in the control arm and 3635 (3873.5) in the intervention arm, which were not significantly different (P = 0.88) (Table 4). The percent change in participant step goals from baseline was not significantly different between study arms (P = 0.49), with a 29% decrease in steps in the control arm and 36% increase in steps in the intervention arm (Table 5). At baseline, the mean (SD) PAR combined stretching and strengthening score was 52.5 (68.9) in the control arm and 47.3 (68.0) in the regret-lottery arm, which also were not significantly different (P = 0.91) (Table 4). The percent change in PAR combined stretching and strengthening score from baseline was not significantly different between study arms (P = 0.84), with a 16% increase in the control arm and 18% increase in the intervention arm (Table 5). At baseline, the mean (SD) PAR aerobic exercise score was 129.0 (150.0) in the control arm and 129.0 (145.4) in the intervention arm on a scale of 0 to 1080, which were not significantly different (P = 0.60) (Table 4). Similarly, the percent change in PAR aerobic exercise score from baseline was not significantly different between study arms (P = 0.55), with a 198% increase in the control arm and a 174% increase in the intervention arm (Table 5).

**Table 4:** Mobility and pain measures at baseline.

Variable	Control, Mean (SD)	Intervention, Mean (SD)	P-value
Daily average no. steps	5907 (3961)	3636 (3873)	0.88
Physical activity			
stretch/strength	52.5 (68.91)	47.3 (68.01)	0.91
aerobic	129.0 (150.0)	129.0 (145.5)	0.60
Chronic pain severity	6.3 (1.52)	7.4 (1.43)	0.81
Chronic pain mobility	24.6 (10.77)	25.4 (10.05)	0.75
Chronic pain activities of daily living	12.2 (12.91)	8.6 (8.56)	0.09
Chronic pain vitality	17.4 (6.11)	19.1 (7.21)	0.69
Chronic pain negative effect	23.4 (13.64)	21.0 (13.28)	0.81
Chronic pain fear	14.9 (4.49)	12.5 (4.76)	0.20
Depression	9.9 (6.08)	9.9 (6.64)	0.74
Daily opioid use Buprenorphine (mg)	30.2 (25.93)	17.2 (8.90)	0.89
Daily opioid use Opioid (MME)	143.3 (227.3)	35.4 (31.54)	0.17

MME- morphine milligram equivalents.

**Table 5:** Percent change in primary and secondary outcomes.

	Control			Intervention			
	Baseline	12 weeks		Baseline	12 weeks		
Variable	Mean (SD)	Mean (SD)	Percent change	Mean (SD)	Mean (SD)	Percent change	P-value
Daily average no. steps	5907 (3961)	4313 (4231)	-29.1	3636 (3873)	4075 (5598)	35.8	0.49
Physical activity							
stretch/strength	52.5 (68.91)	57.0 (60.12)	16.0	47.3 (68.01)	59.3 (61.84)	17.5	0.84
aerobic	129.0 (150.0)	156.0 (116.8)	198.9	129.0 (145.5)	135.8 (123.0)	174.0	0.55
Chronic pain severity	6.3 (1.52)	6.7 (1.63)	10.7	7.4 (1.43)	6.8 (1.95)	-4.7	0.67
Pain Interference							
walking	24.6 (10.77)	25.4 (10.94)	16.2	25.4 (10.05)	24.2 (12.49)	-3.7	0.62
activities of daily living	12.2 (12.91)	15.6 (13.30)	55.3	8.6 (8.56)	8.8 (11.06)	31.4	0.19
vitality	17.4 (6.11)	19.2 (5.32)	29.2	19.1 (7.21)	19.9 (6.37)	17.8	0.87
negative effect	23.4 (13.64)	21.1 (12.11)	17.3	21.0 (13.28)	20.1 (15.39)	-7.0	0.84
fear	14.9 (4.49)	13.1 (4.45)	-6.7	12.5 (4.76)	10.7 (6.77)	-17.0	0.92
Depression (PHQ-9)	9.9 (6.08)	10.7 (6.55)	37.1	9.9 (6.64)	10.0 (6.75)	11.4	0.66
Daily opioid use							
Buprenorphine (mg)	30.2 (25.93)	22.8 (17.65)	-59.1	17.2 (8.90)	21.6 (7.80)	21.7	0.62
Opioid (MME)	143.3 (227.3)	167.2 (244.4)	10.3	35.4 (31.54)	31.9 (35.53)	2.2	0.55

### Chronic pain severity

Veterans reported similar levels of chronic pain at baseline, with those assigned to the control arm reporting a mean (SD) pain score of 6.3 (1.5) and those assigned to the intervention arm reporting a mean (SD) pain score of 7.4 (1.4) (Table 4). Although pain scores increased in the control group and decreased in the intervention group, percent change in pain scores from baseline to 12 weeks did not significantly differ between the two groups (Table 5).

### Pain-related physical function

With respect to pain-related interference, no group differences were noted at baseline with walking, interference with activities of daily living, vitality, negative affect, or fear of reinjury (Table 4). Likewise, the percent change of these various pain-related outcomes over 12 weeks between the control and intervention arms did not statistically differ, although there was a trend for the intervention arm to have greater improvement or less worsening of pain interference scores than the control group (Table 5).

### Depression

With respect to feelings of depression, scores on the PHQ-9 at baseline did not differ between the control (mean = 9.9, SD = 6.08) and intervention (mean = 9.9, SD = 6.64) arms (Table 4). Although not significantly different, symptoms of depression increased by 37% in the control group and 11% in the intervention group over the course of the 12-week study (Table 5).

### Opioid use

Twenty-seven of the 40 participants were taking opioid analgesics at baseline, 15 in the control group and 12 in the regret lottery intervention group. In the control group, nine were using buprenorphine/naloxone and six were taking another opioid, whereas five were using buprenorphine/naloxone and seven were taking another opioid in those assigned to the intervention arm. Baseline buprenorphine/naloxone dose did not differ between the arms, with those in the control group taking a mean (SD) of 30.2 mg (25.9 mg) daily and those in the regret lottery arm taking 17.2 mg (8.9 mg). Likewise, the baseline daily MME doses of opioid containing analgesics did not differ between groups, with those assigned to the control condition taking 143.3mg (227.3mg) and those assigned to the regret lottery taking 35.4mg (31.5mg) (Table 4). No significant group differences were noted in the percent change of buprenorphine/naloxone use or other opioid use over the course of the study (Table 5).

## Discussion

Previous studies have demonstrated the importance of physical activity in decreasing chronic pain and improving functional outcomes [18–22], and behavioral incentives have successfully motivated non-veteran and non-chronic pain patients to engage in increased activity [27–32, 41]. This study represents one of the first to test the use of a regret lottery to motivate veterans with chronic pain to increase their physical activity. The primary objective of this study was to determine if a weekly regret lottery could improve physical activity among veterans with chronic pain, and the secondary objectives were to explore if the lottery intervention would change physical function, chronic pain severity, depression and opioid use.

Despite effectiveness demonstrated in prior studies, this study demonstrated that despite the use of a regret lottery, there were no significant differences appreciated between the intervention and control groups on the primary outcome of physical activity, including step counts, stretching/strengthening and aerobic activity. Further, no group differences were observed for pain-related functional outcomes or chronic pain severity. For the subsample of patients taking opioids, there were no differences in change of medication dose over the course of the study between those in the control condition and those receiving the regret lottery.

There may be multiple reasons for our null findings which differ from those noted in the previous literature. First, the sample was relatively small and not powered for effect, thus positive trends might achieve statistical significance if a larger sample of veterans had been studied. For example, those in receiving the lottery incentive increased the number of steps taken per week by over one-third (35.8%), whereas those in the control arm decreased step count by 29.1%. Similarly, improvements were noted in chronic pain severity, pain interference with mobility and negative affect over 12 weeks for those receiving the lottery incentive, with worsening of the same in the control group. The effect size of behavioral interventions, such as a regret lottery, in the veteran population may be smaller than in other populations (see below), thus a larger sample size may be needed to demonstrate significant effects.

Second, the 3-month follow-up may have been too short to detect significant changes in the primary and secondary outcomes. In particular, decreases in opioid use would be difficult to observe, as dose reductions for patients with longstanding pain typically occur slowly over time. Other studies using similar incentives and use of wearable trackers to increase physical activity took place over periods of 6 months or longer time [29, 43–46].

Third, as noted above, characteristics of the population studied may have restricted the effectiveness of the regret lottery. The study was conducted at a single site at a specialty primary care clinic for veterans with chronic pain. Patients in this clinic had not responded to previous conservative pain management approaches, necessitating referral into a highly resourced, interdisciplinary Pain PACT. Prior behavioral economic approaches that showed success in patients without chronic pain or non-veteran populations may not be generalizable to this study population. Regret lotteries may be more successful for motivating greater physical activity levels for chronic pain patients in general primary care settings or veterans without chronic pain.

### Limitations

There are several potential limitations of our study. Although subjects were blinded to group assignment, it is possible that those in the intervention arm communicated their potential to receive payment to those in the control arm who did not have the same benefit; this may have served as a disincentive for those in the latter group to engage in physical activity since it was not tied to the lottery. Subjects in both groups received large numbers of text messages over the 12-week study period. It is possible that subjects found the messaging to become burdensome or overwhelming over time and thus stopped reading or responding to them as anticipated.

However, this study has several strengths. First, this was a randomized controlled trial permitting causal inferences, necessary when studying interventions in medically and socially complex patient populations. Second, the regret lottery was a simple intervention that could be scaled or modified for other clinics where patients have text-message capable phones. Finally, informal feedback from participants in both arms suggested that veterans viewed the use of activity tracking devices and participation in the study favorably. Other behavioral economic interventions with different approaches may improve physical activity among patients with chronic pain, within and outside of veteran populations, and are worth testing among veterans.

## Conclusions

This study is one of the first to explore the application of behavioral incentives to promote higher levels of physical activity in veterans with chronic pain. The use of a regret lottery did not significantly increase physical activity levels, improve mood or functional outcomes, or decrease opioid use in this study. These findings provide key lessons and insights should larger or longer-term studies evaluate the use of behavioral economic approaches to increase mobility and reduce pain levels among veterans with chronic pain.

## Supporting information

S1 Table Demographic questionnaire. (DOCX)

S1 Data (CSV)

S1 File (PDF)

S1 Checklist CONSORT 2010 checklist of information to include when reporting a randomised trial*. (DOC)
