# Supplementary material for: A randomized controlled trial to evaluate a behavioral economic strategy for improving mobility in veterans with chronic pain
Source: PLoS One. 2021 Oct 11;16(10):e0257320. doi: 10.1371/journal.pone.0257320 (PMC8504760; doi:10.1371/journal.pone.0257320)
Supplement: S1 Table — (DOCX) [file pone.0257320.s001.docx]

Supplement

Demographic Questionnaire

1. What is your race?
   1. White
   2. Black or African American
   3. Native Hawaiian or other Pacific Islander
   4. Other
2. What is your gender?
   1. Male
   2. Female
3. What is your highest level of education?
   1. High school diploma or equivalency (GED)
   2. Associate degree (junior college)
   3. Technical certificate or degree
   4. Bachelors degree
   5. Masters degree
   6. None of the above (less than high school)
   7. Other
4. What is your marital status?
   1. Married
   2. Living with someone as a couple, but not married
   3. Divorced or separated
   4. Never married
5. What best describes your living situation?
   1. Live alone in your own apartment or house
   2. Live with family members
   3. Live with friends or roommates in an apartment or house
   4. Live in a residential treatment facility
6. What best describes your primary mode of transportation?
   1. Bus, SEPTA, train, or other public transportation
   2. Drive myself
   3. A shared van service
   4. Driven by someone else (i.e., family, friends, or a taxi)
7. What best describes your employment status?
   1. Disabled, not able to work
   2. In school
   3. Retired
   4. Unemployed and not looking for work
   5. Unemployed/laid off/looking for work
   6. Working full-time, 35 or more hours per week
   7. Working part-time, less than 35 hours per week
8. What is your estimated annual income?
   1. $10,000 to $14,999
   2. $15,000 to $19,999
   3. $20,000 to $29,999
   4. $30,000 to $39,999
   5. $40,000 to $49,999
   6. $50,000 to $59,999
   7. Do not know
   8. Less than $5,000
   9. Prefer to not disclose
9. Approximately how long does it take for you to get to clinic?
   1. 15-30 minutes
   2. 31-60 minutes (1 hour)
   3. 61-90 minutes
   4. 91-120 minutes (2 hours)
   5. Less than 15 minutes
10. Do you receive all of your health care at the VA?
    1. No
    2. Yes
11. Do you receive any treatment by doctors outside the VA?
    1. No
    2. Yes
12. How old are you?
13. How many people do you live with?
