# Supplementary material for: A randomized controlled trial to evaluate a behavioral economic strategy for improving mobility in veterans with chronic pain
Source: PLoS One. 2021 Oct 11;16(10):e0257320. doi: 10.1371/journal.pone.0257320 (PMC8504760; doi:10.1371/journal.pone.0257320)
Supplement: S1 File — (PDF) [file pone.0257320.s003.pdf]

Research & Development Committee
Department of Veterans Affairs Medical Center
Research and Development Service
University & Woodland Avenue • Philadelphia, PA 19104 • 215-823-6024 • Fax: 215-823-5171

APPROVAL - Initial Review

Date: July 10, 2018
From: Peter W. Groeneveld, MD, MSc, Chairperson
Investigator: Manik Chhabra, MD
Protocol: Connected Health to Decrease Opioid Use in Patients with Chronic Pain
ID: 01758 Prom#: N/A Protocol#: N/A

The following items were reviewed and approved at the 07/03/2018 meeting:

- Informed Consent Document (05/21/2018; 1)
- Request to Review Research Proposal/Project (05/21/2018)
- Abstract (06/20/2018; 1)
- Waiver of Individual Authorization for Disclosure (05/21/2018; 1)
- Authorization for Release of Protected Health Info (05/21/2018; 1)
- Revocation of Authorization for Release of Health (05/21/2018; 1)
- Attestation to Follow Federal Regulations (05/21/2018; 1)
- Research Staff Form (05/21/2018; 1)
- Protocol Summary Specific for CMCVAMC (05/21/2018; 1)
- Investigator Data Form, Page 18 - CHHABRA
- Privacy and Data Security Plan for Initial Reviews (05/21/2018; 1)
- Training Certificates - M. Chhabra and T. Dicks
- Offsite Storage/Data Form (05/21/2018; 1)
- Data Management and Access Plan (05/21/2018; 1)

The protocol was determined to have the following level of risk:
Minimal

Action/Follow-Up: A motion was made and seconded to approve this study. The committee recommended approval. No committee member had a conflict of interest with this study.

Guidance on Reporting the Loss/Theft of VA Research Data: Please refer to IRB SOP, Appendix Q for information on how to report the loss/theft of VA research data.

Approval by each of the following is required prior to study initiation (unless Exempt):
Human Studies Subcommittee (IRB II) [Approval Granted 06/27/2018]
Research & Development Committee

Human Studies Subcommittee (IRB II)
Department of Veterans Affairs Medical Center
Research and Development Service
3900 Woodland Avenue • Philadelphia, PA 19104 • 215-823-3003 • Fax: 215-823-5171

IRB APPROVAL - Initial Review

Date: July 3, 2018
From: Terri M. Laufer, M.D., Chairperson
Investigator: Manik Chhabra, MD
Protocol: Connected Health to Decrease Opioid Use in Patients with Chronic Pain
ID: 01758 Prom#: 0001 Protocol#: N/A

The following items were reviewed and approved through Expedited Review:
- Protocol Summary Specific for CMCVAMC (07/03/2018; 2)

Expedited Approval [Expedited under Federal Regulation: 45 CFR 46.110(b)(1)(4) / VA Regulation: 38 CFR 16.110(b)(1)(4)] was granted on 06/27/2018 for a period of 12 months and will expire on 06/26/2019. Your Continuing Review is scheduled for 05/15/2019. This Expedited review will be reported to the fully convened Human Studies Subcommittee (IRB II) on 06/27/2018.

I. Action/Stipulation: Action by the IRB 2 Chair and the Co-Reviewer of the Month; (no action is required by the IRB): Approved this study (upon stipulations being met) for 12 months at a minimal risk level. It was determined that this research could be reviewed via the Expedited Review Process (45CFR 46110 and 21 CFR 56.110). This study meets Expedited Review, Category 4: Collection of data through noninvasive procedures (not involving general anesthesia or sedation) routinely employed in clinical practice, excluding procedures involving x-rays or microwaves. Where medical devices are employed, they must be cleared/approved for marketing. (Studies intended to evaluate the safety and effectiveness of the medical device are not generally eligible for expedited review, including studies of cleared medical devices for new indications.) Examples: (a) physical sensors that are applied either to the surface of the body or at a distance and do not involve input of significant amounts of energy into the subject or an invasion of the subject's privacy. (b) weighing or testing sensory acuity; (c) magnetic resonance imaging: (d) electrocardiography, electroencephalography, thermography, detection of naturally occurring radioactivity, electroretinography, ultrasound, diagnostic infrared imaging, Doppler blood flow, and echocardiography; (e) moderate excise, muscular strength testing, body composition assessment, and flexibility testing where appropriate given the age, weight and health of the individual.

II. Funding: Institute for Translational Medicine and Therapeutics/Center for Health Incentives & Behavioral Economics at University of Pennsylvania

III. Objective: The primary aim of this pilot study is to determine whether behavioral incentives

The Corporal Michael J. Crescenz VAMC IRB is not connected with, has no authority over, and is not responsible for human research conducted at any other institution, except where a Memorandum of Understanding specifies otherwise. Separate consent forms, initial reviews, continuing reviews, amendments, and reporting of serious adverse events are required if the same study is conducted at multiple institutions.

CMCVAMC SPECIFIC PROTOCOL SUMMARY
Corporal Michael J. Crescenz Department of Veterans Affairs Medical Center (CMCVAMC) Institutional Review Board (IRB)

Section 1. General Information

Protocol Title: Connected Health to Decrease Opioid Use in Patients with Chronic Pain

CMCVAMC Protocol Version Number and Date: Version 2, 7/3/2018

Approved by CMCVAMC IRB 2
07/03/2018

Principal Investigator (PI) Name: Manik Chhabra, MD

PI's Academic Degree(s): *MD*

Is the study funded? *YES* **If "yes", specify funding agency:** *U Penn Institute for Translational Medicine and Therapeutics*

Is a grant application requesting funds for the study currently being reviewed? *NO*

CMCVAMC is the only institution involved: *YES*

CMCVAMC is the coordinating center in which the PI is the lead investigator: *NO*
If this answer is yes, complete the next two sections:

- **List the name(s) of the other site(s) involved.**
- **Provide the FederalWide Assurance (FWA) numbers for each site.**

State name of coordinating center if this is <u>not</u> CMCVAMC.

Describe PI's qualifications to conduct this project, and attach a copy of PI's VA or NIH biosketch. Be specific in regard to PI's research experience. Manik Chhabra, MD is the Principal Investigator (PI) and a staff physician at the CMCVAMC in Philadelphia. He will be entering into the role of Medical Director of the Pain PACT whereupon this study will take place . He previously was a VA Advanced Research Fellow, and a fellow in the Robert Wood Johnson Clinical Scholars Program. He has experience in health services research, clinical trial design and analysis, and statistical analysis. He currently spends 50% of his effort on research and evaluation, and 50% on clinical and teaching activities.

Does any research staff member have an actual and/or perceived conflict of interest with this study? *NO* If yes, explain.

Is this study a clinical trial? *YES* **If yes, specify the type.** *Phase II*

State the estimated length of time to complete enrollment of subjects. *6-9 months*

State the expected duration of participation by individual subjects (including any follow-up, e.g., need to re-contact subject for follow-up questions prior to closure of the study). *12 weeks*

Specify the projected date of completion of the study. *05/31/2019*

Section 2: Participating Site Specifications

2.1. **Where will the research project be conducted? (Check all that apply)**

- ☐ **VA Inpatient Setting**
- XX **VA Outpatient Clinic/Office**
- ☐ **VA Laboratories**
- **Subject Homes**
- ☐ **University of Pennsylvania**
- ☐ **Community Based Outpatient Clinics (CBOCs)**
- ☐ **Other *(Specify)*:** ☐

2.2. **If research is conducted at a non-VA site, please specify where and how much of the project will be conducted at that location.**

Section 3: Introduction

Provide scientific background and rationale for study. Including summary of gaps in current knowledge, relevant data, and how the study will add to existing knowledge. Chronic pain is a highly prevalent and costly condition in the US. An estimated 88.5 million adults suffer from daily pain, resulting in estimated cost of $500-635 billion due to lost productivity, and $261-300 billion in health care expenditures. To manage their chronic pain, 5 to 8 million Americans take an opioid medication daily. Yet, the risks associated with ongoing opioid prescription, including overdose, abuse and diversion, temper their analgesic effects. Opioids are not more effective in the treatment of chronic pain compared with non-opioid approaches. Current guidelines have adapted to the evidence, recommending opioid-sparing approaches for treating patients with chronic pain, and tapering for those on higher doses to safer levels of use. Tapering opioids, however, requires replacing them with effective non-opioid strategies. Improving mobility has been shown to improve pain and decrease medication use among patients chronically prescribed opiates. Concurrently, financial incentives and the use of behavioral incentives have been shown to promote mobility.

Appreciating the gains in health outcomes that can be made with "connected health" approaches, we propose a novel pilot study designed to evaluate if technology enabled care (TEC) strategies and financial incentives can improve patient mobility in our chronic pain population, reduce pain and decrease opioid use. Our primary aim is to determine if chronic pain patients who receive TEC-enhanced treatment with financial incentives demonstrate increased participation in activities that promote mobility (physical therapy, yoga, tai chi) in comparison to patients receiving usual care. Secondary outcomes will include whether increased activity participation also reduces pain severity and opioid use, and improves function and increases the number of daily steps taken. The results of this pilot will enable us to determine what strategies are effective at increasing mobility and if these gains translate into reduced pain and decreased opioid use. The Way to Health application, a research information technology platform at the University of Pennsylvania, will be used to track medication use.

Section 4: Objectives Section

4.1. **Describe the study's purpose, specific aims, or objectives.**

The objective of this pilot study to evaluate if behavioral incentives applied in a VA Medical Center pain-focused patient-centered primary care program (P-PACT) setting can appreciably increase participation in activities that promote mobility, and subsequently reduce pain severity and opioid use.

Primary outcome: Activity participation (as measured by the Stanford Exercise Questionnaire) and increased mobility (as measured by wearable tracker).

Secondary outcomes: Opioid use (as measured by medication adherence and pill counts), physical function (as measured by PROMIS pain interference tool) and pain severity (as measured by PROMIS pain severity tool).

4.2. **State the hypotheses to be tested.**

Hypothesis: Behavioral financial incentives will lead to increased physical activity (as measured by the Stanford Exercise Questionnaire) and mobility (as measured by wearable tracker).

Section 5: Study Procedures

5.1. **Study Design**

Investigative team: Our interdisciplinary team includes a wealth of experience, including nationally known researchers in chronic pain (Compton/Wiedemer), as well as the medical director of the new Pain-PACT (Chhabra). In addition, our team boasts expertise in connected health interventions using behavioral incentives (Chaiyachati).

Study Timeline. Study start-up procedures, including IRB approval and development of text messaging intervention, will take place during the first 3 months of the funding period. Subject recruitment will begin in month 4 and end in month 7, with data collection completed by study month 10. Months 11 and 12 will be devoted to data analysis and dissemination activities.

5.1.2. **What research methods will be used in the project? *Check all that apply.***

X Surveys/Questionnaires — **Interviews** — **Audio Taping**

Behavioral Observations — **Chart Reviews** — **Video Taping**

Focus Groups — **X Randomization** — **Double-Blind**

X Control Group — **Placebo** — **Withhold/Delay Treatment**

Specimen Collection

Deception — **Telephone Survey**

XX Other (Describe) Mobility tracking via a wearable device.

5.1.3. **Provide description of the study population (delineate all categories of subjects – male, female, inpatients, outpatients, providers, family members, employees, etc.). Include anticipated initial enrollment numbers (and number of subjects anticipated to complete all aspects of the protocol).**

The study population will include 40 participants total: 1) adults age 18 years or older; 2) Eligible for P-PACT program; 3) On high dose opioid therapy (MED > 100mg); 4) Possession of activated cell phone with text messaging capabilities; and 5)willingness to comply with study requirements.

5.1.4. **As applicable, provide rationale and information on any added protections and safeguards for vulnerable populations (children, prisoners, pregnant women, physically or mentally-disabled persons, and economically or educationally disadvantaged persons).**

This will not include children or prisoners. For other vulnerable populations, this study will only include subjects wishing to participate. Those individuals with sensory impairments precluding use of text messaging and activity tracker, or with physical disability precluding improvements in physical activity will not be included in the study.

5.1.5. Does this project target a specific race or ethnic group as subjects? *NOT APPLICABLE*

If yes, check all that apply.

**Race**	**Ethnicity**
**American Indian or Alaska Native**	**Hispanic or Latino**
**Asian**	**Not Hispanic or Latino**
**Black or African American**	
**Native Hawaiian or other Pacific Islander**	
**White**	
**Other**	

5.1.6. Will this study bank/store specimens for future research? *NO*

5.1.6.1. **If yes, include information on specimens to be banked/stored.**

5.1.6.2. **If specimens will be banked/stored, specify location.**

5.1.6.3. **If the location of the specimen bank is a <u>non-VA site</u>, has the mandatory approval from VA Central Office been obtained through submission of a tissue banking application?** *Choose an item.*

5.1.6.3.1. **If yes, provide a copy of the response from VA Central Office.**

5.1.6.3.2. ***<u>IF BANKING SPECIMENS, IT MUST BE AT A VA APPROVED FACILITY</u>. (For additional information, go to the following website <u>http://www.research.va.gov/programs/tissue_banking/</u>, or contact the IRB office.)***

5.1.6.4. **If applicable, explain how destruction of banked samples will be substantiated.**

5.1.6.5. **Do you anticipate using the banked specimens for other studies beyond the defined study period and defined study parameters?** *Choose an item.*

5.1.6.5.1. **If yes, will you need to re-contact subjects? How will this be done?**

5.1.7. **Will this study create a <u>data repository for future studies</u>?** *NO*

5.1.7.1. **If yes, describe and/or provide the following:**

5.1.7.1.1. **The type of data (identified or de-identified) including what protected health elements are to be collected.**

5.1.7.1.2. **The source from which data will be collected (e.g., subjects, non-research data repositories, research data repositories, publicly available, VA source, non-VA source.**

5.1.7.1.3. **How and where the data will be stored (e.g., electronic, paper records, approved VA-owned or VA-leased space).**

5.1.7.1.4. **How the data will be transmitted, if applicable.**

5.1.7.1.5. **How the data will be secured during storage, use, and transmission both during the conduct of the research protocol and after the protocol is completed.**

5.1.7.1.6. **Plans to store data for future research. If the data is stored for future research, there must be a description of a research data repository, its location, and its security measures.**

5.1.7.1.7. **Plans to share with others including other researchers (VA and non-VA). If the data were collected through a research project, discussion of whether or not the original informed consent allowed for such reuse of the data and if the reuse is consistent with the HIPAA authorization that was obtained.**

5.1.7.1.8. **Justification for the use of any identifiers.**

5.1.7.1.9. **Justification that the data requested represent the minimum necessary to conduct the research.**

5.1.7.1.10. **A discussion of plans for obtaining informed consent and HIPAA Authorization, or for requesting the IRB to waive these requirements. If the investigator requests that the requirement for a HIPAA Authorization be waived, justification for this request must be included in information submitted to the IRB.**

☐

5.1.7.1.11. **In addition to the above, provide a Standard Operating Procedures Manual for the data repository.** *Contact IRB office for additional details.*

5.2. Subject Recruitment Methods

5.2.1. **State how many subjects will be needed**: 40 total subjects, with 20 in each arm of the study.

5.2.2. **Who will be responsible for recruiting potential subjects? Provide titles of individuals.**
The study team will include:
Manik Chhabra, MD – Principal Investigator
Nancy Wiedemer, RN, MSN, CRNP - Co-Investigator
TBH – Project Manager (hiring in process)

5.2.3. **How will initial contact with potential subjects be made? (*e.g., local clinics, physician referrals, letters to prospective subjects)***
Veterans enrolling in the CMCVAMC P-PACT will be informed of the opportunity to participate in the study by Dr. Chabbra or Ms. Wiedemer, and provided with a copy of the informed consent document to review, and study contact information. The study team will call the veteran 7 days provision of materials. If an individual is interested in the study, they will be asked to review the informed consent and be given the opportunity to ask questions. Once a signed informed consent is returned to the study team, a visit with the veteran will be scheduled to conducted eligibility and baseline surveys. The study team will provide the veteran a wearable device to track step counts and provide instructions to create an account by which they will transmit de-identified step data to the research team and how to access and use the WTH application.

5.2.4. **Will you be using any of the following methods to recruit subjects? (Check all that apply.)**

☐ **Local database for which subjects have NOT given prior permission to be contacted for Research.**

XX☐ **Personal contact with patients over whom you have direct/indirect oversight**

☐ **Provider (Clinician) Referrals of potential subjects**

5.2.5. **Indicate the types of recruitment/advertisement materials that will be used: Check all that apply. Submit copies of recruitment materials, for IRB review.**
X **Not applicable; none to be used**

☐**Fliers** ☐**Newspapers** ☐**Letters** ☐**Websites** ☐**Television**

☐**Radio** ☐**Audio** ☐**Video** ☐**Surveys**

☐**Other (Specify, e.g. employee newsletters)** ☐

5.2.6. **Non-Veteran Subjects will be given a copy of the Notice of Privacy Practices.** *NOT APPLICABLE*

5.3. **Compensation for Participation** - *YES* **If yes, complete the following.**

5.3.1. **Summarize any financial compensation that will be offered to subjects.**

All participants will receive $75 to for completing the entire 12-week study. As noted above, they also have the possibility of receiving a financial incentive via a lottery process for meeting activity goals.

5.3.2. **Provide the schedule for compensation.**

5.3.2.1. **Per study visit or session.**

N/A

5.3.2.2. **Total amount for entire participation.**

$75 if subject completes the entire 12-week study.

5.3.3. **State how compensation will be provided:** *Voucher*

5.4. **Informed Consent Procedures**

5.4.1. **Indicate if informed consent will be obtained and/or if you are requesting a waiver of informed consent or waiver of documentation of informed consent.** *Consent to be obtained*

5.4.2. **If the research involves multiple phases, specify for which phases of the research the waiver(s) is/are being requested.**

N/A

5.4.3. **Describe circumstances, if any, that may need to be addressed in seeking informed consent (e.g., subjects with impaired decision making ability and the use of a legally authorized representative, etc.)**

Not applicable.

5.4.4. **If applicable, indicate how study personnel will be trained regarding human subjects protections requirements and how to obtain and document informed consent.**

All study personnel will have completed the required human subjects, HIPAA, and information security and privacy trainings at the VA.

5.4.5. **Inclusion/Exclusion Criteria: Describe the criteria that determine who will be included in or excluded from the study.**

5.4.5.1. **Inclusion Criteria**

1) adults age 18 years or older; 2) Eligible for P-PACT program; 3) On high dose opioid therapy (MED > 100mg); 4) Possession of activated cell phone with text messaging capabilities; and 5) willingness to comply with study requirements.

5.4.5.2. **Exclusion Criteria**

1) Pain of malignant origin; 2) Sensory impairments precluding use of text messaging and activity tracker; or 3) Physical disability precluding improvements in physical activity.

5.5. **Withdrawal of Subjects**

5.5.1. **Describe how a subject can withdraw from the study.**

Subjects may withdraw at any point by informing the study team in person, by written mail or by phone.

5.5.2. **Describe any anticipated circumstances under which subjects will be withdrawn from the research without their consent.**

None.

5.5.3. **Describe the consequences of a subject's decision to withdraw from the research and the procedures for orderly termination of participation by the subject (e.g., the subject contacting the investigator for an end-of-study visit).**
Patients choosing to withdraw early in the study will not receive the total compensation as described above, but will receive pro-rated payment of $6/wk for each week completed. Patients are free to, at any time, contact the study team to be removed from the study.

5.6. <u>Potential Risk/Benefit Analysis</u>

5.6.1. **Potential Study Risks**

5.6.1.1. **Describe and assess all of the following risks that may be associated with the research:**

5.6.1.2. **Physical**
To minimize the chance for serious and unexpected adverse events, study participants will be screened through exclusion criteria for any health conditions that may be exacerbated by participating in a physical activity study. The program will use a gradual increase in physical activity during the first month that should pose little health risk to participants. Participants are given guidance on when to seek medical attention and a reporting protocol is in place to capture any changes in symptoms with physical activity.

5.6.1.3. **Psychological:** Not applicable.

5.6.1.4. **Social/Economic:** Not applicable

5.6.1.5. **Legal:** Not applicable.

5.6.1.6. **Loss of Confidentiality**
A potential risk of this study is a breach of participant confidentiality. We will minimize this risk by using secure data methods as described previously. Names and addresses will be stored in encrypted databases. These data will be viewable only by the respective participants and the study coordinator. All other members of the research team will be able to view only participant ID numbers. There will be no functionality in the web application to export a dataset with identifiable information. Even the study arms will be identified by code letters until both the statistician and PI agree that analysis is complete.

5.6.1.7. **Other, e.g. radiation, placebo, washout of medications:** Not applicable

5.6.1.8. **Assess the likelihood and seriousness of such risks.**: Not applicable

5.6.2. **Include a description of how anticipated risk will be minimized and include an analysis of risk vs. potential benefit.**
Anticipated risks of this study should be minimal and the risk/benefit ratio is very favorable. To minimize the chance for serious and unexpected adverse events, study participants will be screened through exclusion criteria for any health conditions that may be exacerbated by participating in this study. We have previously outlined the procedures that will be used to prevent a breach of participant data.

5.6.3. **Potential Study Benefits**

5.6.3.1. **Indicate potential benefits to be gained by the individual subjects, as well as benefit(s) that may accrue to society in general as a result of the planned work. If the subject will not receive any direct benefit, this fact must be stated here and in the consent form.**
Through participation in this study, each participant will have the potential to increase physical activity which could improve their health and reduce their opioid use. If this approach is effective, it could have tremendous benefits for society if adopted on a wide scale to help individuals. It is expected that other people will gain knowledge from this study and that

participation could help understand how to effectively motivate individuals to change behavior. Participants may also receive no benefit from their participation in the study.

5.6.4. **Alternative Treatments Outside the Study**

5.6.4.1. **Describe alternatives available to the subject outside the research context. If there are no such alternatives, state that the alternative is not to participate in the research study.**

The alternative is not to participate in the research study.

5.7. **Data Monitoring**

5.7.1. **Will a Data and Safety Monitoring Board (DSMB) or Data Monitoring Committee (DMC) oversee the project?** *NO*

5.7.1.1. **If yes, provide contact information for the DSMB or DMC representative.**

5.7.1.2. **If no, describe the data and safety monitoring plan to be followed.**

The Principal Investigator will be responsible for monitoring the study. All participants will be given anticipatory guidance on when to seek medical attention. In addition, participants will be asked to report to the study team any injuries or medical care that they feel resulted from participation in the study. They can either present in person, call the study team or send an email. The research coordinator will call the participant to collect information regarding the issue and then the PI will review and determine whether it is ok to proceed, further investigation is needed, or the participant should stop the study. For this study, there will be no stopping rules or endpoints and thus no planned interim analyses.

5.8. **Reporting of Protocol Deviations, Adverse Events (AEs), Serious Adverse Events (SAEs), Breaches of Confidentiality, Unanticipated Adverse Device Effects (UADEs), and Unanticipated/Unexpected Problems**

5.8.1. **Include procedures for reporting these events to the CMCVAMC IRB and sponsor.**

Standard protocol will be followed for any events including reporting to the CMCVAMC IRB within 5 business days of discovery. We will use the CMCVAMC serious-adverse event form for reporting SAEs, UADEs, and any other unanticipated/unexpected problems. We will also use the CMCVAMC Protocol Deviation form for reporting any protocol deviations. Any true adverse events will be reported immediately.

5.9. **Privacy and Confidentiality**

5.9.1. **Describe whether the study will use or disclose subjects' Protected Health Information (PHI).**

In order to provide subjects with compensation for enrollment, the study will collect PHI. No PHI will be disclosed to any person outside of the research team.

5.9.2. **Check the PHI to be collected on all subjects for this research protocol.**

- X **Name**
- X **All geographic subdivisions smaller than a State, including street address, city, county, precinct, ZIP code, and their equivalent geographical codes, except for the initial three digits of a ZIP code if, according to the current publicly available data from the Bureau of the Census:**
  - a. **The geographic unit formed by combining all ZIP Codes with the same three initial digits contains more than 20,000 people; and**
  - b. **The initial three digits of a ZIP Code for all such geographic units containing 20,000 or fewer people are changed to 000.**
- X **All elements of dates (except year) for dates directly related to an individual, including birth date, admission date, discharge date, date of death; and all ages over 89 and all elements of dates (including year) indicative of such age, except that such ages and elements may be aggregated into a single category of age 90 or older.**

X **Telephone numbers** ☐ **Fax numbers**
X **Electronic mail addresses** X **Social Security/Medical Record Number**
☐ **Health plan beneficiary numbers** ☐ **Account Numbers**
☐ **Certificate/license numbers**
☐ **Vehicle identifiers and serial numbers, including license plate numbers**
☐ **Device identifiers and serial numbers**
☐ **Web universal resource locators (URLS)**
☐ **Internet protocol (IP) address numbers**
☐ **Biometric identifiers, including fingerprints and voiceprints**
☐ **Full-face photographic images and any comparable images**
☐ **Any other unique identifying number, characteristic, or code, unless otherwise permitted by the Privacy Rule for re-identification.**
☐ **HIV (testing or infectious disease) records** ☐ **Sickle cell anemia**
☐ **Drug Abuse Information** ☐ **Alcoholism or Alcohol Use**

5.10. <u>**Information Security**</u>

5.10.1. **List the data/information that will be stored (including signed, original informed consent and HIPAA authorization forms, if applicable, case report forms, etc.)**
Stored data and information will include: patient information including name, last 4 of SSN, date of birth, BMI, medical, problem list, signed, original informed consent forms, baseline questionnaires capturing participants' sociodemographic information, technology assessment, and current level of physical activity, any responses from follow-up phone interviews.

5.10.2. **Describe the steps that will be taken to secure the data (e.g., training, authorization of access, password protection, encryption, physical controls, Certificates of Confidentiality and separation of identifiers and data).**
All study personnel will complete the required human subjects, HIPAA, and information security and privacy trainings at the VA. Each Veteran who enrolls in this study will be assigned a unique, random patient ID number generated for the purposes of this study. To protect each participant's identity, the link between the Veteran's name, last 4 of SSN, date of birth, and patient ID number will be a password protected file stored on a secure VA server and accessible only to research staff. In all subsequently created analytical files, participants will only be identified by their patient ID number, without inclusion of his/her name, SSN, or date of birth. Subject questionnaires will be either directly inputted into a computer database or written onto paper forms and then transferred to a database at a later time. Interview transcripts will be produced electronically and will be housed on a secure VA server as well. No results will be reported in a personally identifiable manner.

5.10.3. **Indicate how and where data/information will be stored, and specify pertinent security systems.**
The file linking Veterans' personal identifying information, patient database, questionnaire data, and interview transcripts will be password protected and stored on a secure VA server located within the VA firewall. The server is physically located within the FITS computer room of the Philadelphia VAMC and networked within the VA Intranet. Thus, the servers have the same degree of physical and electronic protection afforded other VA computer systems, including

antiviral protection and routine back-ups. FITS is responsible for managing the server hardware and software, including its physical and network security and connectivity, backup processes, operating system patches, and application management. Study data will be accessed using password protected computers that are not connected to the Internet and are entirely compliant with Federal Information Security Management Act (FISMA) standards. Paper records will be kept in a locked file cabinet in an electronically secured building. The likelihood of loss of confidentiality is very low given the information security and privacy requirements that are in place.

The study will use the "*Way to Health*" (WTH) platform to provide close monitoring, feedback and reinforcement at a low cost to permit cost-effective flexible, scalable infrastructure. This platform has been used for clinical trials at the CMCVAMC in the past. The platform was built at the University of Pennsylvania and aims to improve health behaviors and consists of a portal with links to variety of peripheral devices (e.g., scales, wearable devices, glucometers) for assessing health behaviors and outcomes; the capacity to communicate back to patients using interactive voice recording; and the ability to automate the delivery of feedback reports. For this study, medication use and step goal data will be collected via the platform.

Once patients have consented to be in the study and have their data managed by WTH, the WTH platform adherence tracking information will be stored according to a unique, random, patient identifier generated for the purposes of the study. To assure that subject, physician and other informant confidentiality is preserved, individual identifiers (such as name and medical record number) are stored in a single password protected system that is accessible only to study research, analysis and IT staff. This system is hosted onsite at the University of Pennsylvania (UPenn) and is protected by a secure identification number (ID). Any datasets and computer files that leave the firewall will be stripped of all identifiers and individuals will be referred to by their study ID. The study ID will also be used on all analytical files.

The University of Pennsylvania Biomedical Informatics Consortium (BMIC) is the hub for the hardware and database infrastructure. The data collected for WTH based studies is stored in MySQL databases on a BMIC-operated blade server environment devoted specifically to WTH. The data center is housed in the Information Systems and Computing at 3401 Walnut Street. All data are stored in a single relational database, allowing researchers to correct mistakes. Every SQL transaction, including accessing and changing data is logged for auditing purposes. Data are entered into the database through several different mechanisms. A program specialist will enter subjects'unique, random patient identifier and responses to survey questions through a PHP-based web interface (which is based at the BMIC, as noted above). Data from monitoring devices are uploaded automatically. Datasets are blinded of all personally identifiable information when exported for analysis. The web application automatically removes all identifiers when a member of the research team requests an analytic dataset. The only people with access to identifiable participant information are pre-specified Research Coordinators responsible for contacting participants. Personal information and research data will be stored in separate SQL tables and will be linked by a computer-generated ID number. All data for this project will be stored on the secure/firewalled servers for the BMIC Data Center, in data files that will be protected by multiple password layers. These data servers are maintained in a guarded facility behind several locked doors, with very limited physical access rights. They are also cyber-protected by extensive firewalls and multiple layers of communication encryption. Electronic access rights are carefully controlled by UPenn system managers. We believe this multi-layer system of data security, identical to the system protecting the University of Pennsylvania Health System medical records, greatly minimizes the risk of loss of privacy.

5.10.4. **Will PHI be transmitted or transported outside of CMCVAMC?** *NOT APPLICABLE*
As noted above, all PHI will be stored on a VA server located behind a VA firewall.

If yes, complete sections 5.10.4.1 through 5.10.4.3, and an Off-site Storage/Transfer of Research Data form. If no, go directly to section 5.11.

5.10.4.1. **Does the informed consent document and Authorization for Use & Release of Individually Identifiable Health Information for Veterans Health Administration (VHA) Research form disclose entities/individuals to which/whom PHI will be transported or transmitted?** *Choose an item.*

5.10.4.2. **Specify entities/individuals outside CMCVAMC to which/whom data will be disclosed, the justification for such disclosure and the authority, and how they will access it.**

5.10.4.3. **List the data/information that will be transmitted or transported, and specify how data will be transported or transmitted from one location to another and how it will be protected during transmission or transportation outside of CMCVAMC.**

5.11. **Data Management Access Plan**

5.11.1. DMAP form **must** be included with all **initial** submissions. The DMAP form can be found on the Research and Development SharePoint site.

5.12. **<u>Communication Plan</u>**

5.12.1. **Include plan for ensuring that the study is conducted according to the IRB-approved protocol.**

All study personnel will meet regularly to ensure that the study is conducted according to the IRB approved protocol. At these meetings, they will discuss unforeseen challenges as they arise and together create a plan for troubleshooting these issues within the confines of the IRB approved protocol.

5.13. **<u>Is this Study Investigating the Use of a Drug or Biological Agent?</u>** *NO* **If yes, complete the rest of this section. <u>If no, go directly to section 6, unless 5.13 applies.</u>**

5.13.1. **Specify if the drug or biological agent is:**

5.13.1.1. **FDA approved:** *Choose an item.*

5.13.1.2. **Used for off-label purposes:** *Choose an item.*

5.13.2. **Include the FDA Investigational New Drug (IND) number for all non-FDA approved and off-label drugs, biological agents or nutritional supplements. If not applicable state, "Not Applicable."**

5.13.3. **Provide all relevant information about the drug, including pre-clinical data.**

5.13.4. **Explain any wash-out periods, rescue medications permitted and any type of medications not permitted while enrolled in the study.**

5.13.5. **Describe blinding and un-blinding procedures.**

5.13.6. **Include the dosage, route of administration, previous use, and the safety and efficacy information on any drug used for research purposes.**

5.13.7. **Describe rationale for the dosage in this study.**

5.13.8. **Justify why the risks are reasonable in relation to anticipated benefits and/or knowledge.**

5.13.9. **Describe where drug preparation will be done.**

5.13.10. **All drugs for CMCVAMC subjects must be dispensed through the VA investigational pharmacy.**

5.13.11. **Describe where the study treatment will be administered.**

5.13.12. **Describe plan for tracking a non-compliant treatment study subject.**

5.13.13. **Describe the process for the storage, security, dispensing and return of an investigational drug.**

5.13.14. **Has this protocol has been submitted to the Medical Center's Pharmacy and Therapeutics Committee?** *Choose an item.*

5.14. **Is this Study Investigating the Use of a Device** - *NOT APPLICABLE* **If yes, complete the rest of this section. If no, go directly to section 6.**

5.14.1. **The Investigational Device Exemption (IDE) number must be submitted for all significant risk devices and if an IDE exists for a non-significant risk device.**

5.14.2. **Significant Risk or Non-significant Risk - If a device is not approved by the FDA, specify whether or not the sponsor has determined this device to be a "significant risk" or "non-significant risk" as defined by the FDA.**

5.14.3. **Provide all relevant information about the device.**

5.14.4. **Describe blinding and un-blinding procedures.**

5.14.5. **Specify if device is:**

5.14.5.1. **FDA approved:** *Choose an item.*

5.14.5.2. **Used for off-label purposes:** *Choose an item.*

5.14.6. **Explain if the investigational device will be delivered and/or stored by the Principal Investigator or Pharmacy Service.**

5.14.7. **Describe the process for the storage, security, dispensing and return of an investigational device.**

☐

5.14.8. **For research involving an investigational device, describe the SOP or plan for device control.**

☐

5.14.9. **Address how the device will be stored in such a way that only research staff associated with the protocol will have access to the device.**

☐

5.14.10. **Describe measures that will be put into place to ensure that the device will only be used in subjects of this research protocol.**

☐

<u>Section 6: Resources and Personnel</u>

6.1. **Include where and by whom the research will be conducted.**
The study will be coordinated out of the CMCVAMC P-PACT. The team includes: Manik Chhabra, MD (Principal Investigator), and Nancy Wiedemer, RN, BSN, CRNP (co-Investigator), and a project manager to be hired.

6.2. **Provide a brief description of each individual's role in the study. Indicate who will have access to protected health information and who will be involved in recruiting subjects; obtaining informed consent; administering survey/interview procedures; and performing data analysis.**
The team includes: The team includes: Manik Chhabra, MD (Principal Investigator), and Nancy Wiedemer, RN, BSN, CRNP (co-Investigator), and a project manager to be hired. These team members will only have access to PHI and will be working collectively to recruit subjects, obtain consents and administer surveys. Dr. Chhabra will perform data analysis.

6.3. **If applicable, provide information on any services that will be performed by contractors, including what is being contracted out and with whom.**
Not applicable.

6.4. **If applicable, provide information on any Memoranda of Understanding (MOUs) or Data Use Agreements (DUAs) that are being entered into, including with whom and for what reason.**
Not applicable.

<u>Section 7: Genetic Testing</u>

7.1. **Does the project involve genetic testing?** *Not Applicable, SKIP TO SECTION 8*

7.2. **Will specimens be kept for future, unspecified use?** *Choose an item.*

7.3. **Will samples be made anonymous to maintain confidentiality?** *Choose an item.* (*<u>If there is a link, it is not anonymous. Coding is not anonymous.</u>*)

7.4. **Will specimens be destroyed after the project-specific use is completed?** *Choose an item.*

7.5. **Will specimens be sold in the future?** *Choose an item.*

7.6. **Will subjects be paid for their specimens now or in the future?** *Choose an item.*

7.7. **Will subjects be informed of the results of the specimen testing?** *Choose an item.*

7.8. **Are there any implications for family members based on specimen testing results?** *Choose an item.*

7.8.1. **If answer to section 7.8 is yes, they may be subjects.**

7.9. **Will subjects be informed of results obtained from their DNA?** Choose an item.

7.10. **Explain if the study is looking for an association between a genetic marker and a specific disease or condition, but at this point it is not clear if the genetic marker has predictive value.**

7.11. **Describe if the study is based on the premise that a link between a genetic marker and a specific disease or condition is such that the marker is clinically useful in predicting the development of that specific disease or condition.**

7.12. **Will the subject be notified of the results and the provision for genetic counseling?** Choose an item.

Section 8: International Research

8.1. **Does this study involve international research?** *NOT APPLICABLE* **If no, go directly to section 9.**

Section 9: Statistical Analysis

9.1. **Include statistical power calculations and the assumptions made in making these calculations.**
Being a pilot study, the enrollment goal of 40 patients was chosen based on feasibility given the time-frame as opposed to formal power analysis.

9.2. **Define plans for data and statistical analysis, including key elements of the statistical plan, stopping rules and endpoints.**
Data from the clinical trial will be analyzed using statistical software in SAS or R. Changes in pain severity, function, opioid analgesic use and physical activity over time will be compared between subjects assigned to each study arm. Regression analyses will be completed to assess if baseline patient characteristics impact the efficacy of the TEC-linked behavioral incentive intervention on key study outcomes.

9.3. **Provide sample size determination and analysis (include anticipated rate of screen failures, study discontinuations, lost to follow-up, etc.)**
See above.

9.4. **Describe how, where and by whom the data will be analyzed.**
The data will be analyzed Dr. Chhabra with the help of a contracted statistician.

Section 10: References

1. Nahin RL. Estimates of pain prevalence and severity in adults: United States, 2012. *J Pain.* 2015;16(8):769–780.
2. Gaskin DJ, Richard P. The economic costs of pain in the United States. Journal of Pain. 2012; 13(8), 715–724. https://doi.org/10.1016/j.jpain.2012.03.009
3. Chou R, Turner JA, Devine EB, Hansen RN, Sullivan SD, Blazina I, et al. The effectiveness and risks of long-term opioid therapy for chronic pain: a systematic review for a National Institutes of Health Pathways to Prevention Workshop. Ann Intern Med. 2015;162:276-86.
4. Bohnert AS, Valenstein M, Bair MJ, Ganoczy D, McCarthy JF, Ilgen MA, et al. Association between opioid prescribing patterns and opioid overdose-related deaths. JAMA 2011;305(13):1315-1321.
5. Dunn KM, Saunders KW, Rutter CM, Banta-Green CJ, Merrill JO, Sullivan MD, et al. Opioid prescriptions for chronic pain and overdose: a cohort study. Ann Intern Med. 2010;152(2):85-92.
6. Boscarino JA, Rukstalis M, Hoffman SN, et al. Risk factors for drug dependence among out-patients on opioid therapy in a large US health-care system. Addiction 2010;105:1776–82.
7. Krebs EE, Gravely A, Nugent S, Jensen AC, DeRonne B, Goldsmith ES, Kroenke K, Bair MJ, Noorbaloochi S. Effect of Opioid vs Nonopioid Medications on Pain-Related Function in Patients With Chronic Back Pain or Hip or Knee Osteoarthritis PainThe SPACE Randomized Clinical Trial. *JAMA.* 2018;319(9):872–882. doi:10.1001/jama.2018.0899

8. Powell D, Haegerich TM, Chou R. CDC Guideline for Prescribing Opioids for Chronic Pain - United States, 2016. MMWR Recomm Rep. 2016 Mar 18;65(1):1-49.
9. Department of Veterans Affairs/Department of Defense. Management of opioid therapy for chronic pain. https://www.va.gov/painmanagement/docs/cpg_opioidtherapy_summary.pdf
10. Chou R, Huffman LH. Nonpharmacologic therapies for acute and chronic low back pain: A review of the evidence for an American Pain Society/American College of Physicians clinical practice guideline. Ann Intern Med. 2007; https://doi.org/10.7326/0003-4819-147-7-200710020-00007
11. Saper RB, Lemaster C, Delitto A, Sherman KJ, Herman PM, Sadikova E., ... Weinberg J. Yoga, physical therapy, or education for chronic low back pain: A randomized noninferiority trial. Ann Intern Med. 2017; 167(2), 85–94. https://doi.org/10.7326/M16-2579.
12. Wieland LS, Skoetz N, Pilkington K, Vempati R, D'Adamo CR, Berman BM. Yoga treatment for chronic non-specific low back pain. Cochrane Database of Systematic Reviews. 2017; https://doi.org/10.1002/14651858.CD010671.pub2
13. Wang C, Iversen MD, McAlindon T, Harvey WF, Wong JB, Fielding RA, ... Schmid CH. Assessing the comparative effectiveness of Tai Chi versus physical therapy for knee osteoarthritis: design and rationale for a randomized trial. BMC Complementary and Alternative Medicine, 2014; 14. https://doi.org/10.1186/1472-6882-14-333
14. Patel, M. S., Benjamin, E. J., Volpp, K. G., Fox, C. S., Small, D. S., Massaro, J. M., ... Murabito, J. M. (2017). Effect of a game-based intervention designed to enhance social incentives to increase physical activity among families: The BE FIT randomized clinical trial. *JAMA Internal Medicine*, *177*(11), 1586–1593. https://doi.org/10.1001/jamainternmed.2017.3458
15. Patel, M. S., Volpp, K. G., Rosin, R., Bellamy, S. L., Small, D. S., Fletcher, M. A., ... Asch, D. A. (2016). A Randomized Trial of Social Comparison Feedback and Financial Incentives to Increase Physical Activity. *American Journal of Health Promotion*, *30*(6), 416–424. https://doi.org/10.1177/0890117116658195
16. Patel MS, Asch DA, Volpp KG. Wearable devices as facilitators, not drivers, of health behavior change. JAMA. 2015 Feb 3;313(5):459-60. doi: 10.1001/jama.2014.14781.
17. Colorafi K. Connected health: a review of the literature. Mhealth. 2016 Apr 14;2:13. doi: 10.21037/mhealth.2016.03.09.
18. Cella, D., Riley, W., Stone, A., Rothrock, N., Reeve, B., Yount, S., ... Hays, R. (2010). The patient-reported outcomes measurement information system (PROMIS) developed and tested its first wave of adult self-reported health outcome item banks: 2005-2008. *Journal of Clinical Epidemiology*, *63*(11), 1179–1194. https://doi.org/10.1016/j.jclinepi.2010.04.011

Waiver or Alteration of Informed Consent Requirements/Waiver of Requirement to Obtain Documentation of Informed Consent
Corporal Michael J. Crescenz Department of Veterans Affairs Medical Center (CMCVAMC) Institutional Review Board (IRB)

**Principal Investigator Name:**	Manik Chhabra, MD
**Title of Study:**	Connected Health to Decrease Opioid Use in Patients with Chronic Pain
**Study ID #: 01758**	**Study Prom #:0001**

☑ **Waiver or Alteration of Informed Consent** [45 CFR 46.116(d)(c); 38 CFR 16.116(d)(c)]
An IRB may approve a consent procedure which **does not include, or which alters**, some or all of the elements of informed consent set forth in this section, or waive the requirements to obtain informed consent provided the IRB finds and documents that: This form is being used for pre-sceening. It does not preclude the Informed Consent process. PI must obtain written informed consent via the ICD and HIPAA forms.

☑ 1. The research involves no more than minimal risk to the subjects;

☑ 2. The waiver or alteration will not adversely affect the rights and welfare of the subjects;

☑ 3. The research could not practicably be carried out without the waiver or alteration; and

☑ 4. Whenever appropriate, the subjects will be provided with additional pertinent information after participation.

☑ 5. The research or demonstration project is to be conducted by or subject to the approval of state or local government officials and is designed to study, evaluate, or otherwise examine:
a. Public benefit or service programs;
b. Procedures for obtaining benefits or services under those programs;
c. Possible changes in or alternatives to those programs or procedures; or
d. Possible changes in methods or levels of payment for benefits or services under those programs.

☐ **Waiver of Requirement to Obtain Documentation of Informed Consent** [45 CFR 46.117(c)(1-2); 38 CFR 16.117(c) This is being used in conjunction with the oral ICD.
An IRB may **waive the requirement for the investigator to obtain a signed consent** form for some or all subjects if it finds either:

☐ 1. That the only record linking the subject and the research would be the consent document and the principal risk would be potential harm resulting from a breach of confidentiality. Each subject will be asked whether the subject wants documentation linking the subject with the research, and the subject's wishes will govern; or

☐ 2. That the research presents no more than minimal risk of harm to subjects and involves no procedures for which written consent is normally required outside of the research context.

☐ *In cases in which the documentation requirement is waived, the IRB may require the investigator to provide subjects with a written statement regarding the research.*n/a

☐ **Waiver or Alteration of Informed Consent** (FDA 21 CFR 50.23)

A. The obtaining of informed consent shall be deemed feasible unless, before use of the test article (except as provided in paragraph (b) of this section), both the investigator and a physician who is not otherwise participating in the clinical investigation certify in writing all of the following:
   1. The human subject is confronted by a life-threatening situation necessitating the use of the test article.
   2. Informed consent cannot be obtained from the subject because of an inability to communicate with, or obtain legally effective consent from, the subject.
   3. Time is not sufficient to obtain consent from the subject's legal representative.
   4. There is available no alternative method of approved or generally recognized therapy that provides an equal or greater likelihood of saving the life of the subject.

B. If immediate use of the test article is, in the investigator's opinion, required to preserve the life of the subject, and time is not sufficient to obtain the independent determination required in paragraph (a) of this section in advance of using the test article, the determinations of the clinical investigator shall be made and, within 5 working days after the use of the article, be reviewed and evaluated in writing by a physician who is not participating in the clinical investigation.

C. The documentation required in paragraph (a) or (b) of this section shall be submitted to the IRB within 5 working days after the use of the test article.

D. D(1) Under 10 U.S.C. 1107(f) the President may waive the prior consent requirement for the administration of an investigational new drug to a member of the armed forces in connection with the member's participation in a particular military operation. The statute specifies that only the President may waive informed consent in this connection and the President may grant such a waiver only if the President determines in writing that obtaining consent: Is not feasible; is contrary to the best interests of the military member; or is not in the interests of national security. The statute further provides that in making a determination to waive prior informed consent on the ground that it is not feasible or the ground that it is contrary to the best interests of the military members involved, the President shall apply the standards and criteria that are set forth in the relevant FDA regulations for a waiver of the prior informed consent requirements of section 505(i)(4) of the Federal Food, Drug, and Cosmetic Act (21 U.S.C. 355(i)(4)).

NOTE: FDA regulation - When deciding if a protocol is FDA regulated, a determination must be made whether or not research results will be submitted to or held for inspection by the FDA. If research results will be submitted to or held for inspection by the FDA, then the research must be considered FDA regulated.

RECOMMENDATION: **APPROVED**

SIGNATURE OF IRB CHAIR/VICE-CHAIR OR DESIGNEE	Date
[signature]	7/3/18

WAIVER OF INDIVIDUAL AUTHORIZATION FOR DISCLOSURE OF PROTECTED HEALTH INFORMATION
Corporal Michael J. Crescenz Department of Veterans Affairs Medical Center (CMCVAMC)
Institutional Review Board (IRB)

**Principal Investigator's Name:**	Manik Chhabra
**Title of Study:**	Connected Health to Decrease Opioid Use in Patients with Chronic Pain
**Waiver Version Date and Version #:**	5/18/2018, VERSION 1

PLEASE PROVIDE THE FOLLOWING INFORMATION:

1. **The use or disclosure of the requested information involves no more than minimal risk to the privacy of individuals based on the following elements (VHA Handbook 1200.05).**

1.1. **Provide an adequate plan to protect the identifiers from improper use and disclosure.**

1.1.1. **If the collected identifiable data will be recorded in such a manner that human subjects can be identified, directly or through identifiers linked to the subjects, or allow re-identification in the future, provide the measures to protect individuals' privacy and confidentiality of data.** Linked identifiable data will used to confirm a Veteran's identity. A randomly generated participant ID will then be created for each participant. This randomly generated ID will be used to follow the participant throughout the study. The participant ID and link to the participant's identifiable data will be stored in a locked room, on a password protected server with access only to the principle investigator and study team. This data will be stored on site at the VA Medical center, 3900 Woodland Avenue, Annex Suite 202, Room 232, which is a typical and secure area to house identifiable data for research studies

1.1.2. **If links to identifiers are used:**

1.1.2.1. **Describe the coding mechanism the research protocol will use.** We will use a random code generator to create a participant ID which will be used throughout the study to track participants.

1.1.2.2. **List the individuals who have access to the coding mechanism.** The principal investigator and research coordinator will have sole access to the coding mechanism

1.2. **Provide an adequate plan to destroy the identifiers at the earliest opportunity consistent with conduct of research, unless there is a health or research justification for retaining the identifiers or such retention is otherwise required by law. Note: All research records, including the investigator's research records, must be retained according to the National Archives and Records Administration VHA's Records Control Schedule.**

Upon completion of the study, identifiers will be removed from the CHERP server. No paper records will be kept of participant identifiers. All data will be kept in a single, on site location and at the end of the study period, all identifying information will be purged from the system.

1.3. **Provide adequate written assurances that the PHI will not be reused or disclosed to any other person or entity, except as required by law, for authorized oversight of the research study, or for other research for which the use or disclosure of the requested information is permitted by the Privacy Rule (VHA Handbook 1200.05).**

1.3.1. **Explain why the research could not practicably be conducted without this waiver.** This research will need to identify potentially eligible veterans who have been identified with chronic pain and have been prescribed 100 morphine miliequivalents or higher. We will also need their address to mail them a letter about this study and their phone number to contact them about their interest.

1.3.2. **Provide an explanation why this research could not practicably be conducted without access to and use of the requested information.** *Without the initial data to identify and recruit eleigible patients, the study team would be unable to perform the study as it could not identify patients to participate.*

1.4. **This waiver of authorization is for: (Check only one of the following)**

1.4.1. **X Use of Protected Health Information (PHI) only for recruitment of study subjects.**

1.4.2. ☐ **Use or disclosure of PHI for recruitment of study subjects and one or more phases or aspects of the study. List/describe the phase or aspects.** ☐

1.4.3. ☐ **Use or disclosure of PHI for one or more phases or aspects of the study but not recruitment. List/describe the phase or aspects.** ☐

2. **Give a brief description of the PHI for which the IRB has determined use or disclosure to be necessary (VHA Handbook 1200.05). (In addition, complete the following HIPAA identifier table.)**

Veteran's phone number and home address.

3. **Check the PHI to be collected on all subjects for this research protocol.**

X Name

X All geographic subdivisions smaller than a State, including street address, city, county, precinct, ZIP code, and their equivalent geographical codes, except for the initial three digits of a ZIP code if, according to the current publicly available data from the Bureau of the Census:
a. The geographic unit formed by combining all ZIP Codes with the same three initial digits contains more than 20,000 people; and
b. The initial three digits of a ZIP Code for all such geographic units containing 20,000 or fewer people are changed to 000.

X All elements of dates (except year) for dates directly related to an individual, including birth date, admission date, discharge date, date of death; and all ages over 89 and all elements of dates (including year) indicative of such age, except that such ages and elements may be aggregated into a single category of age 90 or older.

X Telephone numbers

X Electronic mail addresses

☐ **Health plan beneficiary numbers**

☐ **Certificate/license numbers**

☐ **Vehicle identifiers and serial numbers, including license plate numbers**

☐ **Device identifiers and serial numbers**

☐ **Web universal resource locators (URLs)**

☐ **Internet protocol (IP) address numbers**

☐ **Biometric identifiers, including fingerprints, voiceprints**

☐ **Full-face photographic images and any comparable images**

☐ **Fax numbers**

X Social Security/Medical Record Number

☐ **Account Numbers**

☐ Any other unique identifying number, characteristic, or code, unless otherwise permitted by the Privacy Rule for re-identification.

☐ HIV (testing or infectious disease) records ☐ Sickle cell anemia

☐ Drug Abuse Information ☐ Alcoholism or Alcohol Use

4. **If social security numbers (SSNs) are going to be used, describe the specific use, the type of SSN to be used (real, scrambled, last 4 digits) and the security measures in place for protecting them. Or state, not applicable.**
Initial enrollment will verify a Veteran's identity by name, date of birth and last 4 digits of the social security number. Once identified this information will not be used again.

5. **Indicate what other "specific" health information (past, present, or future physical or mental health or condition of the individual) is required to be used and collected. Or state, not applicable.**

We will ask Veteran's to complete a telephone survey. The survey will gather demographic information, information about their current physical activity and their current use of technology. The Veterans completing the study will also be asked about their physical activity at the end of the study and their perceptions on the technology used. We will use the electronic medical record to obtain information on the veteran's medical conditions.

6. **Indicate the number of charts that could be looked at to obtain the number of patients to be approached.** We plan to enroll 40 participants. We anticipate approximately 20% of participants approached will participate in the study, thus anticipate accessing approximately 200 patient records

7. **Indicate by name and location the databases from which the information will be obtained. Or state, not applicable. All data will be stored on the CHERP servers. Located at 3900 Woodland Avenue, Annex Suite 202, Room 232. The drive location address is: \\VHAPHIFPCCHERP.v04.med.va.gov/shares2**

8. **Investigator's Assurance to Protect the Privacy of Subjects and to Maintain the Confidentiality of Information:**

X I assure the CMCVAMC IRB that the information that I provided in this application is accurate and complete; that the Individual Identifiable Information that I request is the minimum amount of identifiable private information necessary for my research project; and that the identifiable information will not be reused or disclosed to any other person or entity, except (a) as required by law, (b) for authorized oversight of the research study, or (c) for other research for which the use or disclosure of individual identifiable information would be permitted by the HIPAA Privacy Rule.

X I assure the CMCVAMC IRB that the purpose of the data is to conduct scientific research and that no personnel involved in the study may identify, directly or indirectly, any individual patient or subject in any report of such research or otherwise disclose patient or subject identities in any manner.

X I assure the CMCVAMC IRB that only qualified research personnel will have access to the research subjects' identifiable information.

X I and my research staff have completed the VHA Privacy training for this fiscal year.

SIGNATURE OF PRINCIPAL INVESTIGATOR	Date
[signature]	5/18/2018

FOR [illegible] IRB OFFICE USE ONLY BELOW THIS LINE

NOTE: For an IRB or Privacy Board to approve a waiver of HIPAA authorization for research, it must determine that the following criteria have been met as required by 45 CFR 164.512(i)(2) (VHA Handbook 1200.05).

The IRB has determined that the use or disclosure of the requested information involves no more than a minimal risk to the <u>privacy</u> of individuals based on the following elements (check all that apply):
Note: If an IRB determines that all criteria are <u>not met, the IRB cannot approve the waiver.</u>

- [x] **An adequate plan to protect the identifiers from improper use and disclosure.**
- [x] **An adequate plan to destroy the identifiers at the earliest opportunity consistent with conduct of the research, unless there is a health or research justification for retaining the identifiers or such retention is otherwise required by law.**
- [x] **Adequate written assurances that the PHI will not be reused or disclosed to any other person or entity, except as required by law, for authorized oversight of the research study, or for other research for which the use or disclosure of PHI would be permitted by the HIPAA Privacy Rule.**
- [x] **The research could not practicably be conducted without the waiver or alteration.**
- [x] **The research could not practicably be conducted without access to and use of the PHI.**

- [ ] **IRB 1 Registration # IRB00000692**
- [x] **IRB 2 Registration # IRB00003822**

**Study ID #:** 01758	**Study Prom #:** 0001

- [ ] **Approved Full Board Review**
38 CFR 16.108(b); 45 CFR 46.108(b)
- [x] **Approved Expedited Review**
38 CFR 16.110; 45 CFR 46.110

The criteria of this waiver of HIPAA authorization, as provided by the PI, were deemed satisfactory so as to justify the waiver, and the use or disclosure of the requested information involves no more than minimal risk to the <u>privacy</u> of individuals.

- [x] **Use of PHI only for recruitment of study subjects**
- [ ] **Use or disclosure for recruitment of study subjects and one or more phases or aspects of the study**
- [ ] **Use or disclosure for one or more phases or aspects of the study but not recruitment.**

**SIGNATURE OF IRB CHAIR/VICE-CHAIR OR DESIGNEE**	**Date**
[signature]	7/3/18

CATEGORIES QUALIFYING FOR EXPEDITED REVIEW (45 CFR 46.110)
Corporal Michael J. Crescenz Department of Veterans Affairs Medical Center (CMCVAMC) Institutional Review Board (IRB)

**Principal Investigator Name:**	Chhabra, Manik, MD
**Title of Study:**	Connected Health to Decrease Opioid Use in Patients with Chronic Pain
**Study ID #: 01758**	**Study Prom #0001**

☐ **Category 1** - Clinical studies of drugs and medical devices only when condition (a) or (b) is met. (a) Research on drugs for which an investigational new drug application (21 CFR Part 312) is not required. (b) Research on medical devices for which (i) an investigational device exemption application (21 CFR Part 812) is not required; or (ii) the medical device is cleared/approved for marketing and the medical device is being used in accordance with its cleared/approved labeling.

☐ **Category 2** - Collection of blood samples by finger stick, heel stick, ear stick, or venipuncture as follows: (a) from healthy, non-pregnant adults who weigh at least 110 pounds. For these subjects, the amounts drawn may not exceed 550 ml in an 8 week period and collection may not occur more frequently than 2 times per week; or (b) from other adults and children, considering the age, weight, and health of the subjects, the collection procedures, the amount of blood to be collected, and the frequency with which it will be collected. For these subjects, the amount drawn may not exceed the lesser of 50 ml or 3 ml per kg in an 8 week period and collection may not occur more frequently than 2 times per week.

☐ **Category 3** - Prospective collection of biological specimens for research purposes by noninvasive means. Examples: (a) Hair and nail clippings in a nondisfiguring manner; (b) Deciduous teeth at time of exfoliation or if routine patient care indicates a need for extraction; (c) Permanent teeth if routine patient care indicates a need for extraction; (d) Excreta and external secretions (including sweat); and (e) Uncannulated saliva collected either in an unstimulated fashion or stimulated by chewing gumbase or wax or by applying a dilute citric solution to the tongue; (f) Placenta removal at delivery; (g) Amniotic fluid obtained at the time of rupture of the membrane prior to or during labor; (HP Supra- and subgingival dental plaque and calculus, provided the collection procedure is not more invasive than routine prophylactic scaling of the teeth and the process is accomplished in accordance with accepted prophylactic techniques; (i) Mucosal and skin cells collected by buccal scraping or swab, skin swab, or mouth washings; (j) sputum collected after saline mist nebulization.

☑ **Category 4** - Collection of data through noninvasive procedures (not involving general anesthesia or sedation) routinely employed in clinical practice, excluding procedures involving x-rays or microwaves. Where medical devices are employed, they must be cleared/approved for marketing. (Studies intended to evaluate the safety and effectiveness of the medical device are not generally eligible for expedited review, including studies of cleared medical devices for new indications.) Examples: (a) physical sensors that are applied either to the surface of the body or at a distance and do not involve input of significant amounts of energy into the subject or an invasion of the subject's privacy. (b) weighing or testing sensory acuity; (c) magnetic resonance imaging: (d) electrocardiography, electroencephalography, thermography, detection of naturally occurring radioactivity, electroretinography, ultrasound, diagnostic infrared imaging, Doppler blood flow, and echocardiography; (e) moderate excise, muscular strength testing, body composition assessment, and flexibility testing where appropriate given the age, weight and health of the individual.

☐ **Category 5** - Research involving materials (data, documents, records, or specimens) that have been collected for any reason or will be collected solely for non-research purposes (such as medical treatment or diagnosis).

☐ **Category 6** - Collection of data from voice, video, digital, or image recordings made for research purposes.

☐ **Category 7** - Research on group characteristics or behavior (including, but not limited to, research on perception, cognition, motivation, identity, language, communication, cultural beliefs or practices, and social behavior) or research employing survey, interview, oral history, focus group, program evaluation, human factors evaluation, or quality assurance methodologies.

☐ **Category 8** - Continuing review of research previously approved by a convened IRB as follows: (a) where (i) the research is permanently closed to the enrollment of new subjects; (ii) all subjects have completed all research-related interventions; and (iii) the research remains active only for the long term follow-up of subjects; or (b) where no subjects have been enrolled and no additional risks have been identified or (c) where the remaining research activities are limited to data analysis.

- *For a multi-center protocol, an expedited review procedure may be used by the IRB at a particular site whenever the conditions of category (8)(a), (b), or (c) are satisfied for that site. However, with respect to category 8(b), while the criterion that "no subjects have been enrolled" is interpreted to mean that no subjects have ever been enrolled at a particular site, the criterion that "no additional risks have been identified" is interpreted to mean that neither the investigator nor the IRB at a particular site has identified any additional risks from any site or other relevant source.*

☐ **Category 9** - Continuing review of research not conducted under an investigational new drug application or investigational drug exemption where categories 2 through 8 do not apply but the IRB has determined and documented at a convened meeting that the research involves no greater than minimal risk and no additional risks have been identified.

- *The determination that "no additional risks have been identified" does not need to be made by the convened IRB.*

RECOMMENDATION: APPROVED

**Signature of Chair/Vice-Chair or Designee**	**Date**
[signature]	**7/3/2018**

RESEARCH STAFF FORM

Corporal Michael J. Crescenz Department of Veterans Affairs Medical Center Institutional Review Board (CMCVAMC IRB)

***NOTE: Submit required CMCVAMC training for each individual listed on this form** NOTE: If you are using a MAC, you may have difficulty with "choose an item" boxes.

**TITLE OF RESEARCH PROTOCOL:**	**Connected Health to Decrease Opioid Use in Patients with Chronic Pain**	**DATE:07/03/2018**

**ID #:01758**	**PI Name**	**Staff Member Name**	**Staff Member Name**	**Staff Member Name**
*Name	Manik Chhabra	Tanisha Dicks		
VA and/or Other Business Email Address	Manik.chhabra@va.gov	Tanisha.dicks@va.gov		
VA and/or Other Business Telephone Number	612-860-0053	215-823-5800 x7157		
Non-VA Telephone Number	612-860-0053	215-823-5800 x7157		
Role on this protocol, e.g. PI/Co-I; Res. Assistant; Study Coordinator; Consultant	PI	Study Coordinator		
% Effort on Study	**5%**	**20%**		
Is this staff member a VA employee?	**YES**	**YES**	Choose an item.	Choose an item.
Is consenting subjects at CMCVAMC **new** to this staff member? If so, s/he **MUST** take the training offered by the RCO.	**NO**	**NO**	Choose an item.	Choose an item.
Does this staff member exercise independent clinical decision making related to this protocol?	**YES**	**NO**	Choose an item.	Choose an item.
Does this staff member come to the CMCVAMC and see either VA patients or their private health information for this protocol?	**YES**	**NO**	Choose an item.	Choose an item.
Does this staff member decide if potential subjects meet inclusion/exclusion criteria for this protocol?	**YES**	**YES**	Choose an item.	Choose an item.
Does this staff member obtain subjects' consent for this protocol?	**NO**	**YES**	Choose an item.	Choose an item.
Does this staff member document in CPRS for this protocol?	**NO**	**YES**	Choose an item.	Choose an item.

Approved by CMCVAMC IRB 2
07/03/2018

HRPP Accepted: 09/20/2017
Philadelphia (642); Research & Development (151)
